# Magnoliae Cortex Alleviates Muscle Wasting by Modulating M2 Macrophages in a Cisplatin-Induced Sarcopenia Mouse Model

**DOI:** 10.3390/ijms22063188

**Published:** 2021-03-20

**Authors:** Minwoo Hong, Ik-Hwan Han, Ilseob Choi, Nari Cha, Woojin Kim, Sun Kwang Kim, Hyunsu Bae

**Affiliations:** 1 Department of Science in Korean Medicine, College of Korean Medicine, Kyung Hee University, 26-6 Kyungheedae-ro, Dongdaemun-gu, Seoul 02453, Korea; minwoo1939@naver.com (M.H.); dlftjq1308@naver.com (I.C.); chanari1205@naver.com (N.C.); wjkim@khu.ac.kr (W.K.); skkim@khu.ac.kr (S.K.K.); 2 Department of Physiology, College of Korean Medicine, Kyung Hee University, 26-6 Kyungheedae-ro, Dongdaemun-gu, Seoul 02453, Korea; joyful153@naver.com

**Keywords:** sarcopenia, M2 macrophage, Magnoliae Cortex, IGF-1, cisplatin

## Abstract

Cachexia causes high mortality, low quality of life, and rapid weight loss in cancer patients. Sarcopenia, a condition characterized by the loss of muscle, is generally present in cachexia and is associated with inflammation. M2 macrophages, also known as an anti-inflammatory or alternatively activated macrophages, have been shown to play a role in muscle repair. Magnoliae Cortex (M.C) is a widely used medicinal herb in East Asia reported to have a broad range of anti-inflammatory activities; however, the effects of M.C on sarcopenia and on M2 macrophage polarization have to date not been studied. This study was designed to investigate whether the oral administration of M.C could decrease cisplatin-induced sarcopenia by modulating M2 macrophage polarization in mice. C57BL/6 mice were injected intraperitoneally with cisplatin (2.5 mg/kg) to mimic chemotherapy-induced sarcopenia. M.C extract (50, 100, and 200 mg/kg) was administered orally every 3 days (for a total of 12 times). M.C (100 and 200 mg/kg) significantly alleviated the cisplatin-induced loss of body mass, skeletal muscle weight, and grip strength. In addition, M.C increased the expression of M2 macrophage markers, such as MRC1, CD163, TGF-β, and Arg-1, and decreased the expression of M1-specific markers, including NOS2 and TNF-α, in skeletal muscle. Furthermore, the levels of like growth factor-1(IGF-1), as well as the number of M2a and M2c macrophages, significantly increased in skeletal muscle after M.C administration. M.C did not interfere with the anticancer effect of cisplatin in colon cancer. Our results demonstrated that M.C can alleviate cisplatin-induced sarcopenia by increasing the number of M2 macrophages. Therefore, our findings suggest that M.C could be used as an effective therapeutic agent to reverse or prevent cisplatin-induced sarcopenia.

## 1. Introduction

Sarcopenia is defined as a loss of muscle mass and strength and is one of the features of cachexia. This muscle loss is characterized by rapid muscle atrophy and decreased activity, leading to a sharp decline in the quality of life and higher mortality [1,2]. Sarcopenia can be induced by malignancy, as well as by a chemotherapy treatment [3]. Cisplatin is an effective chemotherapy medication commonly used to treat solid tumors; however, it causes severe side effects, including neurotoxicity, nephrotoxicity, ototoxicity, and rapid muscle wasting. The body weight loss in cancer patients treated with cisplatin is mostly due to muscle wasting [4]. Furthermore, it has been shown that inflammatory cytokines, such as TNF-α, play an important role in muscle wasting in sarcopenia [5]. Although sarcopenia causes various adverse outcomes, such as reduced quality of life and higher mortality, effective therapeutic interventions have to date not been developed [6]. The European Working Group on Sarcopenia in Older People (EWGSOP2) also highlights the ever-increasing likelihood of delaying, reversing treatment, and preventing, sarcopenia early in patients [7]. Therefore, new therapies for sarcopenia are urgently needed.

Macrophages play an essential role in the regeneration and growth of the fiber membrane during the repair of muscle injury [8]. M1 macrophages are an important source of TNF-α and cause muscle aging by affecting muscle cell fusion with sarcopenia and aging muscle fibers [9]. M1 macrophages by macrophage polarization produce pro-inflammatory cytokines, such as IL-1, TNF-α, and IL-6, causing muscle wasting and increasing protein degradation via the lysis of muscle fibers [10]. Moreover, in the same muscle sarcopenia model, when TNF-α is administered to mice, muscle regeneration and muscle mass are weakened [11]. However, M2 macrophages secrete several anti-inflammatory cytokines, such as TGF-β and IL-10, leading to muscle recovery and regeneration, and the acceleration of muscle fiber synthesis. IL-10 has been shown to regulate the switch between M1 and M2 macrophages [12]. Furthermore, macrophage-derived IGF-1 shifts polarization towards M2a and M2c phenotypes, the macrophage populations involved in extracellular matrix remodeling and muscle healing, promoting muscle recovery and protecting from muscle dystrophy [13,14]. Therefore, the balance between M1 and M2 macrophage populations is critical for muscle recovery [15].

Prescriptions containing *Magnolia officinalis Rehder et Wilson* (Magnoliae Cortex) are still used in modern clinical practice in Asian countries [16]. Magnoliae Cortex (M.C) has been reported to have anti-inflammatory and anti-oxidant activities and to inhibit nitric oxide (NO) production in lipopolysaccharide (LPS)-activated macrophages [17,18]. At the same time, in vitro and in vivo studies have demonstrated that the M.C extract has no genotoxic or mutagenic properties [19]. Furthermore, due to its anti-inflammatory properties, M.C has been used as a dietary supplement, as well as cosmetic and periodontal treatment [20,21]. Magnolol and honokiol, the major components of M.C, are reported to have anti-inflammatory, anti-oxidant, anti-cancer, anti-diabetes, anti-microbial, anti-stress, and other biological properties [22]. Previous studies have shown that magnolol suppresses muscle protein degradation, inhibits inflammatory responses, and increases IGF-1-mediated protein synthesis [8]. In addition, honokiol has been reported to inhibit the LPS-induced TNF-α secretion in macrophages and to exhibit anti-cancer activity [23]. However, it is not known whether M.C, which contains magnolol and honokiol, has a therapeutic effect on sarcopenia.

Therefore, we evaluated the effect of M.C extract on muscle wasting using a mouse model of cisplatin-induced sarcopenia. We also investigated changes in macrophage polarization and IGF-1 expression in response to the treatment with the M.C extract. Finally, we assessed the effect of M.C treatment on cisplatin-induced tumor formation.

## 2. Results

### 2.1. Identification of Active Components in M.C Using HPLC

**Figure 1:**
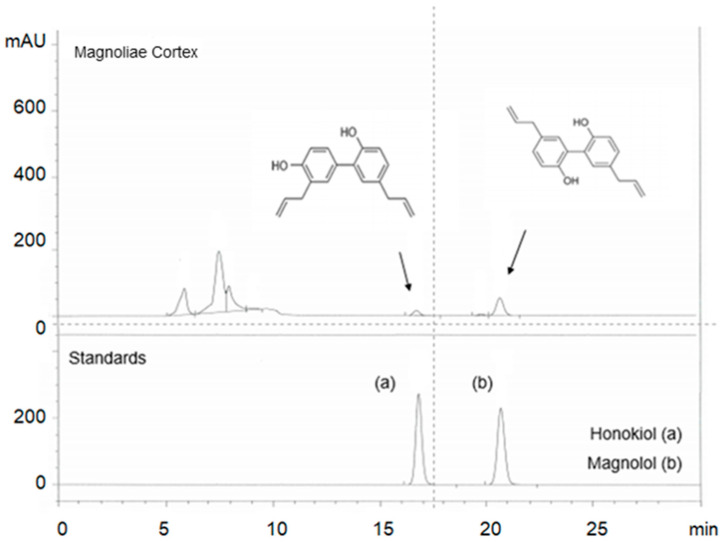
Identification of the active components in Magnoliae by Cortex using HPLC. Two major compounds of Magnoliae Cortex (M.C), honokiol (0.8%) and magnolol (2.1%), were identified using HPLC by comparing the retention time and UV spectra of the standard compounds.

First, we verified the presence of two major compounds in M.C, honokiol and magnolol, by comparing the retention time and UV spectra of standard compounds using the established high pressure liquid chromatography (HPLC) protocol (Figure 1). The presence of honokiol and magnolol was confirmed and was determined to be approximately 0.5% and 2.1% of M.C, respectively.

### 2.2. M.C Decreased Cisplatin-Mediated Weight Loss in Mice

**Figure 2:**
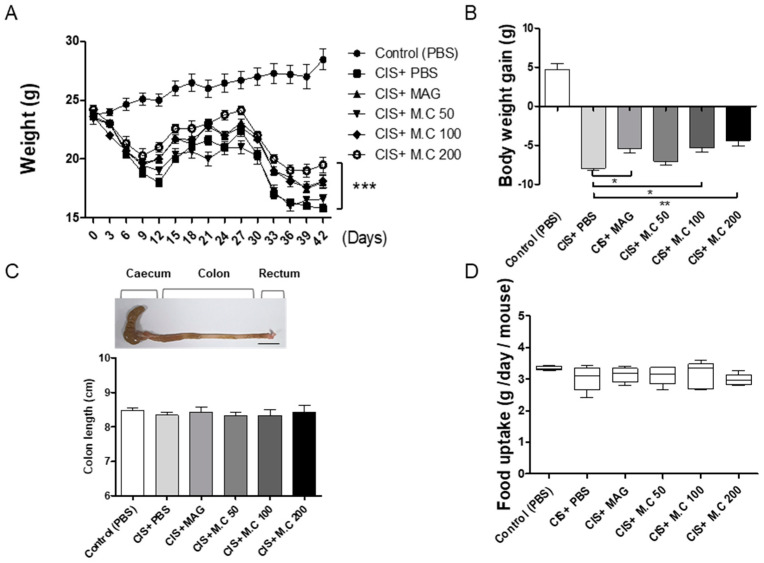
The effect of M.C treatment on body weight loss and dietary intake measurements in mice with cisplatin-induced sarcopenia. To induce muscle damage, cisplatin (2.5 mg/kg) was administered on days 1–5 and 26–30. Magnolol (10 mg/kg) was administered intraperitoneally every 3 days, for a total of 12 times. M.C (50, 100, and 200 mg/kg) was administered orally every 3 days, for a total of 12 times. Mice were sacrificed on day 42: (**A**) weight changes in the sarcopenia mouse model. Data are presented as the mean ± SEM (*n* = 10/group). * *p* < 0.05, ** *p* < 0.01, *** *p* < 0.001 vs. cisplatin, using a Bonferroni post hoc test after two-way ANOVA; (**B**) weight changes in the sarcopenia mouse model presented as % vs. control (42 days); (**C**) representative colon images (scale bar 1 cm) and colon length measurement; (**D**) average daily feed intake per mouse. Data are presented as the mean ± SEM (*n* = 10/group). * *p* < 0.05, ** *p* < 0.01, *** *p* < 0.001 vs. cisplatin (CIS)+phosphate-buffered saline (PBS), using a Bonferroni post hoc test after one-way ANOVA.

Cisplatin, a common chemotherapy medication, causes rapid weight loss in mice. Mice were injected intraperitoneally daily with cisplatin (2.5 mg/kg) on days 1–5 and 26–30. Magnolol (10 mg/kg) was administered intraperitoneally every 3 days (for a total of 12 times) and was used as a positive control [24]. M.C (50, 100, and 200 mg/kg) was administered orally every 3 days (for a total of 12 times). Phosphate-buffered saline (PBS) was administered orally every 3 days. As expected, the cisplatin injection resulted in body weight loss in mice; however, the administration of M.C and magnolol alleviated cisplatin-induced weight loss (Figure 2). The induction of weight loss with cisplatin showed a rapid decrease and a partial recovery starting on day 15; however, there was no recovery after the second dose of cisplatin. At the same time, the groups treated with magnolol and M.C (100 and 200 mg/kg) showed faster body weight recovery on days 12 and 39 compared to the cisplatin group. This experiment was ended on day 42, as significant difference between the cisplatin (CIS)+PBS and CIS+M.C groups persisted for more than 6 days, and the weight of the mouse decreased by more than 30% (Figure 2A). Then, we compared the weight change between the last day and the first day. Mice treated with cisplatin demonstrated a reduction in body weight compared to the control group, while magnolol and M.C significantly decreased the cisplatin-induced weight loss (Figure 2B). To determine whether body weight loss was associated with digestion and the lack of appetite, the colon length and average daily food intake were measured (Figure 2C,D). Our results demonstrated that there were no differences in colon length and food consumption between groups, suggesting that cisplatin-induced body weight was not associated with abnormal digestion or appetite changes.

### 2.3. M.C Alleviated Muscle Wasting in Skeletal Muscle

**Figure 3:**
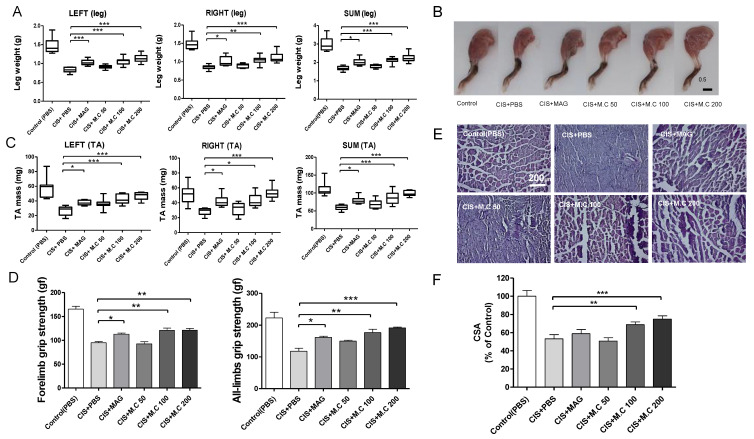
M.C alleviated muscle wasting induced by the cisplatin treatment in mice. The effect on muscle was evaluated by measuring leg mass, TA (tibialis anterior) muscle mass, fiber diameter, and grip strength. Data were obtained from the mouse leg tissue on day 42: (**A**) leg muscle mass; (**B**) representative leg images (scale bar 0.5 cm); (**C**) TA muscle mass; (**D**) The grip strength was measured using behavioral tests in front legs and in all legs; (**E**,**F**) hematoxylin and eosin (H&E) staining of TA muscle (scale bar 200 μm) and cross-sectional area (CSA) analysis. Data are presented as the mean ± SEM (*n* = 10/group in A–C, *n* = 5/group in E–D). * *p* < 0.05, ** *p* < 0.01, *** *p* < 0.001 vs. CIS+PBS, using a Bonferroni post hoc test after one-way ANOVA.

Then, we evaluated the effect of cisplatin on skeletal muscle wasting by measuring the leg weight and tibialis anterior muscle (TA) muscle mass. Leg weight measurements were conducted on day 42 and representative leg images are shown in Figure 3A,B. The TA muscle was measured as a representative skeletal muscle (Figure 3C). Data showed that both magnolol and M.C had alleviated the loss of cisplatin-induced leg weight and muscle mass. To determine the effect of cisplatin on grip strength, leg muscle strength was measured (Figure 3D). As expected, the pulling force power was increased in magnolol and M.C (100 and 200 mg/kg) groups compared to the cisplatin group; however, there was no significant effect in the 50 mg/kg M.C group. To evaluate muscle fiber damage, the muscle fiber shape in the skeletal muscle was assessed by the hematoxylin and eosin (H&E) staining (Figure 3E). M.C (100 and 200 mg/kg) prevented muscle fiber deformation, whereas 50 mg/kg M.C failed to prevent muscle fiber damage. Therefore, the treatment with M.C (100 and 200 mg/kg) and magnolol prevented muscle loss and damage in mice with cisplatin-induced sarcopenia; however, the 50 mg/kg M.C dose did not have any significant effect.

### 2.4. M.C Affected M1 and M2 Macrophage Polarization in Skeletal Muscle

**Figure 4:**
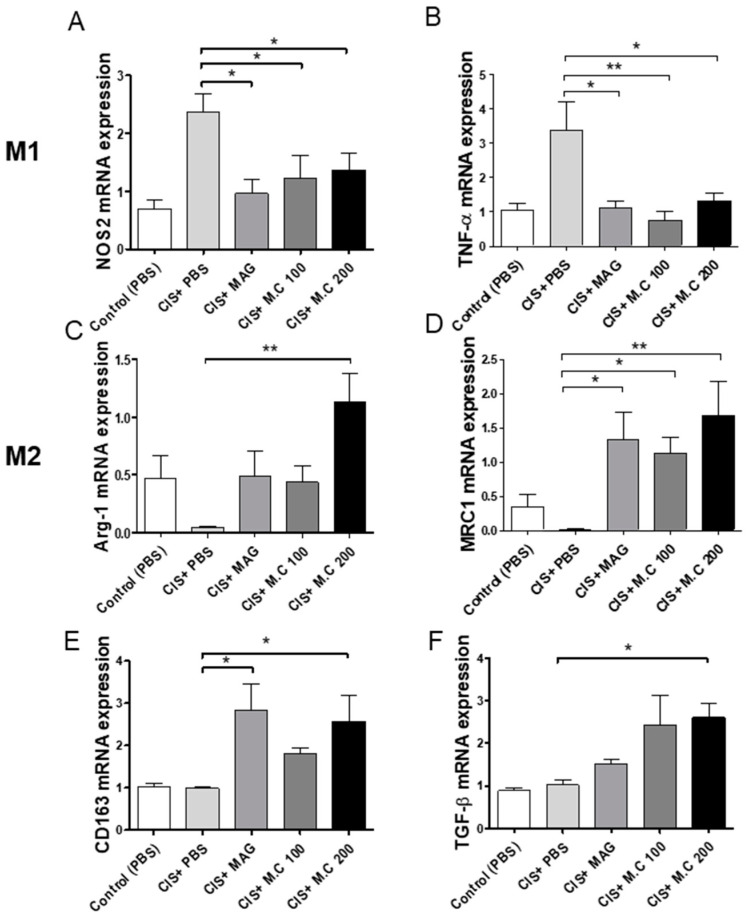
M.C modulated M1 and M2 macrophage polarization in skeletal muscle. The mRNA expression of M1- and M2-specific genes was assessed by qPCR: (**A**) NOS2; (**B**) TNF-α; (**C**) Arg-1; (**D**) MRC1; (**E**) CD163; and (**F**) TGF-β. M1 macrophage makers (**A**,**B**), and M2 macrophage makers (**C**–**F**). Data are presented as the mean ± SEM (*n* = 4~5/group). * *p* < 0.05, ** *p* < 0.01 vs. CIS+PBS, using a Bonferroni post hoc test after one-way ANOVA.

The repair of damaged muscle fibers requires the activation of M2 macrophages [25]. Therefore, the gene expression of macrophage-specific markers, including Arg-1, NOS2, TNF-α, MRC1, TGF-β, and CD163, was determined in skeletal muscle by real-time quantitative PCR (qPCR) (Figure 4). Our results demonstrated that the expression of M1 markers (TNF-α and iNOS) was increased in the cisplatin-treated group; however, the M.C treatment decreased their expression (Figure 4A,B). At the same time, the levels of M2 markers (CD206, Arg-1, TGF-β, and CD163) were upregulated by the M.C treatment compared to the cisplatin group (Figure 4C–F). These data suggested that M.C modulates M1 and M2 macrophage polarization in the skeletal muscle microenvironment.

### 2.5. The Number of Macrophages and the Expression of IGF-1 Increased in Skeletal Muscle after M.C Treatment

**Figure 5:**
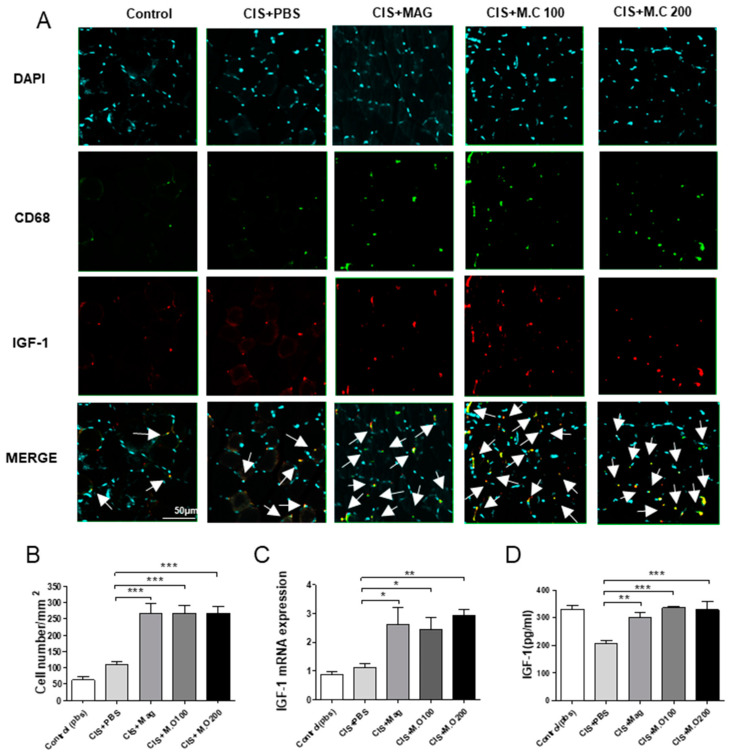
*M.C* increased the number of macrophages and the expression of IGF-1 in the TA muscle: (**A**) Immunohistochemistry (IHC) showing CD68 (macrophages, green) and IGF-1 (red) staining in the TA muscle. (Magnification: 40×, scale bar 50 μm). Nuclei are stained with diamidino-2-phenylindole (DAPI) (blue). Merge = IGF-1 + CD68 + DAPI and merged places are indicated by white arrows. (**B**) The number of IGF-I, CD68, and DAPI positive cells per image area measured on at least five random fields. (**C**) IGF-1 mRNA expression in the TA muscle tissue measured by qPCR. (**D**) IGF-1 protein expression in the TA muscle tissue was quantified using ELISA. Data are presented as the mean ± SEM (*n* = 4~5/group). * *p* < 0.05, ** *p* < 0.01, *** *p* < 0.001 vs. CIS+PBS, using a Bonferroni post hoc test after one-way ANOVA.

Then, we investigated the macrophage expression of IGF-1 in mice with muscle damage. The skeletal muscle sections were stained for CD68 (a macrophage marker), IGF-1, and counterstained with diamidino-2-phenylindole (DAPI) (Figure 5A). The number of cells positive for CD68 and IGF-1 from five random fields of the skeletal muscle was quantified. Our results demonstrated that the number of cells expressing both CD68 and IGF-1 was increased in M.C and magnolol treatment groups (Figure 5B). Furthermore, both mRNA and the protein expression of IGF-1 were also significantly increased in M.C treatment groups compared to the cisplatin group (Figure 5C,D), confirming our previous observations.

### 2.6. M1, M2a, and M2c Macrophage Subtypes Changed after the M.C Treatment

**Figure 6:**
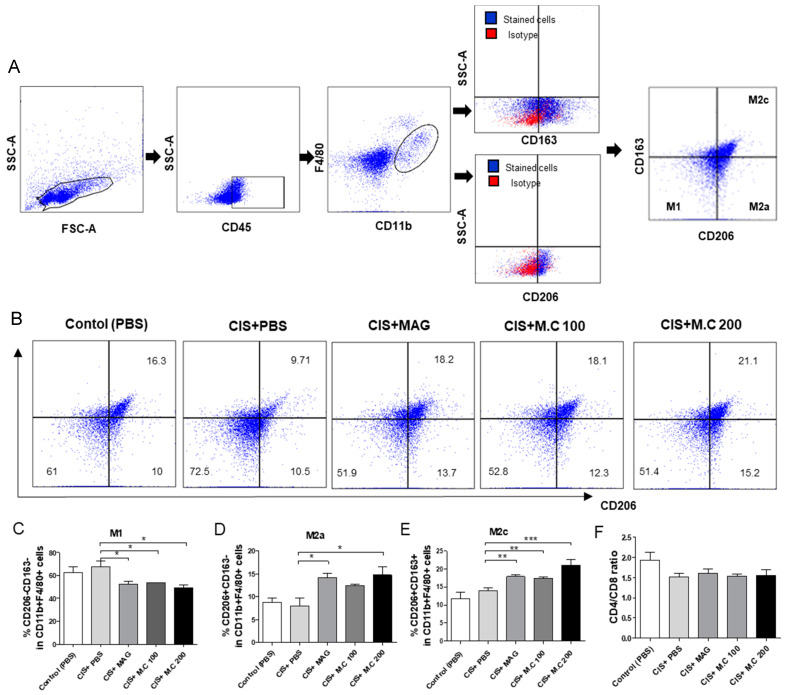
M.C increased the ratio of M2a and M2c macrophages. Spleen-derived cells from the sarcopenia mouse model were assessed by flow cytometry. (**A**,**B**) Representative flow cytometry (FACS) dot plots of CD206, CD163 in CD11b^+^F4/80^+^, and CD45^+^cells. CD206^−^CD163^−^ M1 macrophages; CD206^+^CD163^−^ M2a macrophages; CD206^+^CD163^+^M2c macrophages; (**C**–**E**) graphs of M1 macrophage, M2a macrophage, and M2c macrophage, presented as % of CD11b^+^F4/80^+^ cells; (**F**) the CD4^+^/CD8 ratio of CD45^+^ cells in splenocytes, presented as the mean ± SEM (*n* = 4/group). * *p* < 0.05, ** *p* < 0.01, *** *p* < 0.001 vs. CIS+PBS, using a Bonferroni post hoc test after one-way ANOVA.

To investigate the effects of M.C on M1 macrophage phenotype changes, we used spleen-derived macrophages. Cell phenotypes were defined as follows: M1 macrophages as CD206^−^CD163^−^ cells, M2a macrophages as CD206^+^CD163^−^ cells, M2c macrophages as CD206^+^CD163^+^ cells, gated on CD45^+^ and CD11b^+^F4/80^+^ cells (Figure 6A). Our results showed that the *M.C treatment significantly decreased* CD206^−^CD163^−^ *M1 populations within* CD11b^+^F4/80^+^ *macrophages* compared to the cisplatin treatment group (Figure 6C). In addition, the M2a macrophage (CD206^+^CD163^−^) and M2c macrophage (CD206^+^CD163^+^) populations were significantly increased in response to the magnolol and M.C treatment (Figure 6D,E). The CD4^+^/CD8^+^ T cell ratio indicating changes in T-cell subsets was also measured and there were no differences between the groups (Figure 6F).

### 2.7. M.C Did Not Obstruct the Anti-Tumor Activity of Cisplatin

**Figure 7:**
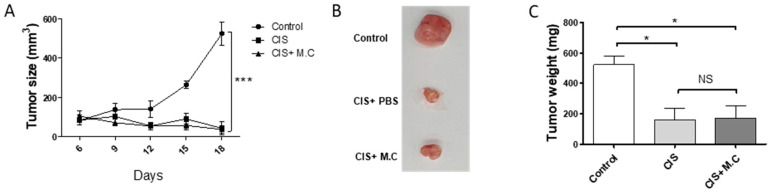
M.C does not interfere with cisplatin activity in tumors. CT26 tumor subcutaneous injections were administered in control, cisplatin (CIS), CIS+M.C (cisplatin+ M.C) treated mice. (**A**) Tumor size. Tumor volumes measured every 3 days using a caliper. Data are presented as the mean ± SEM (*n* = 5). * *p* < 0.05, *** *p* < 0.001 vs. control, using a two-way ANOVA. Mice were sacrificed on day 18; (**B**) representative images of the tumors; (**C**) tumor weight measurement. Data are presented as the mean ± SEM (*n* = 4/group). * *p* < 0.05, *** *p* < 0.001 vs. CIS+PBS, using a Bonferroni post hoc test after one-way ANOVA. NS = non-significant.

Finally, we tested whether the M.C treatment interfered with the anti-tumor activity of cisplatin, a common chemotherapy medication. In the murine colon carcinoma cell line (CT26) colon cancer-bearing mouse model, cisplatin significantly reduced tumor growth (Figure 7). Moreover, although there was no significant difference in the mouse colon cancer model that showed a tendency toward a faster weight gain recovery period than the cisplatin alone treatment, when treated with M.C (Supplementary Figure S2). Furthermore, the M.C treatment did not have any effect on the cisplatin-induced decrease in tumor size and weight. These results suggested that the M.C treatment would not interfere with the chemotherapeutic functions of cisplatin.

## 3. Discussion

Cisplatin is the most common chemotherapeutic medication for the treatment of solid cancers. Unfortunately, cisplatin has been reported to have several adverse effects, including muscle atrophy. Therefore, the mitigation of cisplatin-induced cachexia would decrease one of the major side effects of chemotherapy. M.C is commonly used in the traditional clinical practice because of its anti-inflammatory properties; however, its effect on macrophage polarization, as well as on sarcopenia, has not been investigated yet. In this study, we demonstrated that the M.C extract (100 and 200 mg/kg) treatment can effectively alleviate cisplatin-induced muscle atrophy and body weight loss. Furthermore, that M2 macrophages and IGF-1 expression could be modulated by M.C treatment. The cisplatin group measured a higher proportion of pro-inflammatory M1 macrophages in skeletal muscle, whereas the M.C treatment improved the M1/M2 imbalance.

A previous study by Chen et al. demonstrated that magnolol attenuated the skeletal muscle atrophy and reduced the levels of inflammatory cytokines, such as IL-1β, TNF-α, and IL-6, in muscle tissue and serum [26]. Here, in this study, we presented evidence showing that the extract of M.C and its major ingredient, magnolol, alleviated muscle-wasting sarcopenia induced by cisplatin, and that this effect could be associated with the modulation of macrophage polarization. For example, cisplatin reduced mouse body weight, while the M.C treatment partially recovered that weight loss (25–30% of the vehicle-treated control group) (Figure 2). A grip strength test was used to assess muscle strength loss. And muscle wasting in skeletal muscle was measured by weight and H&E (Figure 3). Furthermore, the extract of M.C, as well as magnolol, also significantly alleviated cisplatin-induced muscle wasting. Therefore, these results suggested that the ethanol extract of M.C improved sarcopenia, with magnolol being the major active ingredient.

We also investigated the effect of M.C treatment on macrophages (Figure 4 and Figure 6, Supplementary Figure S1), since these cells are known to play an essential role in muscle repair in mice. Furthermore, a reduction or depletion of macrophages at the injured site has been shown to impair skeletal muscle regeneration after injury [27]. M1 macrophages inhibit muscle fiber fusion by releasing TNF-α and IL-1β, while M2 macrophages promote muscle fiber formation by releasing anti-inflammatory cytokines, such as TGF-β and IL-10, and increasing the expression of IGF-1 [28]. IGF-1 in the effect muscle protein synthesis has been received [9]. Previous reports indicated that the muscle damaged in sarcopenia was importantly related to the downregulation of p70S6K/mTOR/4EBP1 pathway [11]. Proinflammatory cytokines and myostatin are able of impairing IGF-1 signaling and IGF-1 bioavailability [29,30]. Moreover, IGF-1 is capable of triggering Akt-induced FoxO subsequent degradation and phosphorylation [31]. Comprehensively, it has been reported that IGF-1 is suggested to increase protein synthesis. Therefore, a new therapeutic strategy for the treatment of sarcopenia could involve the alleviation of inflammation progression by controlling the balance between M1 and M2 macrophages in the muscle.

The M2 macrophage phenotype, depending on the state of stimulation and transcription, can be further divided into M2a and M2c subtypes [32]. The M2a subtype expresses Arg-1 and is associated with anti-inflammatory cytokines and with muscle repair [33]. The M2c subtype is activated by IL-10 and, in turn, secretes IL-10, resulting in immune suppression and tissue remodeling. M2a and M2c macrophages express high levels of matrix metalloproteinase-8 (MMP-8), CD163, and CD206, the proteins connected to angiogenesis and matrix remodeling [34]. M.C has been reported to have various biological activities, including anti-inflammatory, antioxidant, anti-cancer, anti-diabetes, antimicrobial, and anti-stress properties [22]. Major phytochemicals isolated from M.C include phenolic compounds, alkaloids, and neolignan derivatives [35]. In our previous experiment, a low dose of magnolol (1 mg/kg) significantly attenuated cisplatin-induced sarcopenia [24]. Although the route of administration was different (intraperitoneal vs. oral), this result demonstrated that magnolol may be the major active component in the effect of M.C against muscle wasting. Furthermore, honokiol are reported to show anti-inflammatory, anti-oxidant and anti-cancer effects [36]. Thus, based on these results, Magnolol (5,5′-di-2-propenyl-1,1′-biphenyl-2,2′-diol) and honokiol (3′,5-di-2-propenyl-1,1′-biphenyl-2,4′-diol), neolignan derivatives with similar chemical structures, may be considered as the two principal phenolic compounds in M.C [20,37]. In macrophages, magnolol blocks p38 kinase, suppressing NF-κB/Rel signaling associated with M1 activation. NF-κB is a major transcription factor related to inhibit macrophage M1 activation. Magnolol, known to suppress muscle protein degradation, inhibit inflammatory responses, and increase IGF-1-mediated protein synthesis, is a promising supplement for preventing chemotherapy-induced skeletal muscle atrophy associated with cancer-related cachexia [8]. We observed that the treatment with the Magnoliae extract induced a high expression of IGF-1. In the sarcopenia mouse model, M2c macrophages have been associated with recovering muscle [24]. Here, we show that the magnolol treatment increased the levels of both M2c and M2a macrophages. These results suggest that, in addition to magnolol, other components present in the M.C extract may play a role in improving muscle repair. Honokiol is another main ingredient of M.C, and is also expected to alleviate sarcopenia for three reasons: (1) honokiol has the ability to cross the blood–brain barrier and has high oral bioavailability; (2) honokiol inhibits NO and TNF-α expression, as well as PKCα, NF-κB, and MAPK signaling pathways [36]; and (3) honokiol saves skeletal muscle wasting by inhibiting NF-κB inflammation and induced oxidative stress [7]. Therefore, honokiol could play an important anti-inflammatory role in vivo [23]; in addition, when honokiol and magnolol are co-administered, skeletal muscle increases glucose absorption by activating Akt in a pl3k-dependent manner, which is a key element of the insulin signaling pathway in L6 myotubes [30]. Therefore, these two synergies are expected to have a positive effect on sarcopenia. However, further studies are necessary to clarify the role of honokiol in muscle wasting.

## 4. Materials and Methods

### 4.1. Animal and Cell Line

Mice (C57BL/6, 7–8 weeks old) were obtained from the Jackson Laboratory (Raonbio, Gyeonggi, Korea). Animals were randomly caged and housed in a regulated environment with a 12 h light and dark cycle and free access to food and water. All experimental protocols were approved by the Kyung Hee Institutional Animal Care (KHUASP (SE)-20-529). The CT26 murine colon carcinoma cell line was purchased from the Korean Cell Line Bank (80009; Seoul, Korea). The cell line was cultured in DMEM (D5671; Welgene, Gyeongsangbuk, Korea) supplemented with 100 U/mL penicillin, 100 μg/mL streptomycin (Invitrogen Life Technologies, Rockville, MD, USA), and 10% heat-inactivated fetal bovine serum (S001-01; Welgene, Gyeongsangbuk, Korea). Cells were sub-cultured every three days. Cells were maintained in an incubator at 37 °C, 5% CO_2_, and 95% humidity for all experiments. CT26 tumor cells (3 × 10^5^ cells per mouse) were injected subcutaneously into mice. All efforts were made to reduce animal suffering during the experiments.

### 4.2. Chemicals

Magnolol (purity ≥ 95%) was obtained from Sigma (m3445; Sigma-Aldrich, St. Louis, MO, USA). Magnolol was dissolved in DMSO to prepare 10 mM stock solution. Cisplatin was purchased from Sigma-Aldrich (P4394; St. Louis, MO, USA) and dissolved in saline at 1 mg/mL.

### 4.3. The Preparation of M.C

M.C *(Magnolia officinalis Rehder et Wilson*, 200 g, Sunchen, Korea) was prepared using a mixed solvent of ethanol: water (7:3, *v*/*v*) as follows: 1000 mL of the solvent was added to 200 g of M.C, extracted in a reflux system at 70 °C, repeated twice, and then filtered. The 70% ethanol extract of M.C was concentrated under reduced pressure at 40–45 °C and freeze-dried for 3 days. The percent yield of the freeze-dried M.C was 10.91% (21.82 g). The components of M.C were analyzed by HPLC. Magnolol and honokiol present in M.C, as well as standards, were analyzed by Dong-eul Herbal Medicine Analysis Center (Busan, Korea). The octadecyl silica (ODS)-chromatographic analysis of magnolol and honokiol was performed using a DHAC-12-01 (Agilent, Santa Clara, CA, USA) equipped with a UV–vis spectrophotometer (289 nm). Magnolol and honokiol were analyzed using an OSAKASODA C18 column (5 um particle size, 4.6 × 250 mm; Osaka, Japan) at 20 °C by isocratic elution using acetonitrile: distilled water: acetic acid (70:30:1, *v*/*v*/*v*) as mobile phase components. The injection volume and flow rate were 10 µL and 0.3 mL/min, respectively. Magnolol and honokiol standards were detected at 289 nm for the qualitative HPLC analysis. M.C powder (0.1 g) was dissolved in 70% methanol (100 mL), sonicated for 20 min, filtered, and then used for the ODS-HPLC analysis. Magnolol and honokiol standards were dissolved in 100% methanol at a concentration of 100 mg/L for the HPLC analysis. M.C was tested for the main components, magnolol and honokiol. Magnolol (2.1%) and honokiol (0.8%) were detected.

### 4.4. Flow Cytometry

Spleen samples were dissolved in 40 μm nylon mesh strainer. Cells of red blood were lysed as Pharmlyse buffer (555899; BD bioscience, San Jose, CA, USA). The following antibodies were used: anti-mCD8 APC-CY7 (100713; BioLegend, San Diego, CA, USA), anti-mCD4 APC (100412; BioLegend, San Diego, CA, USA), anti-mCD45 Pacific Blue (MCD4528; Invitrogen, Carlsbad, CA, USA), anti-mF4/80 BV421 (123131; BioLegend, San Diego, CA, USA), anti-mCD11b PerCP-Cy 5.5 (101227; BioLegend, San Diego, CA, USA), anti-mCD163 PE (12-1631-82; e-bioscience; Thermo Fisher Scientific, Middlesex County, MA, USA), and anti-mCD206 APC (141707; BioLegend, San Diego, CA, USA). Samples were washed in flow cytometer (FACS) buffer, followed by incubation for 1 h. The cells were washed twice and resuspended in FACS buffer for the analysis using BD FACS Lyric instruments (BD bioscience, San Jose, CA, USA). Data were analyzed using FlowJo V10 software (Treestar Inc., San Carlos, CA, USA). Unstained cells (negative controls) were used as gating controls.

### 4.5. Histology and Immunohistochemistry Analysis

TA muscle tissues were fixed for 24 h in 4% formalin, embedded in paraffin, cut into 4 μm sections, processed for histology, stained with hematoxylin and eosin (H&E) to evaluate the pathological changes in the tissues as previously described by Lee et al., and imaged using an Eclipse Ci-L microscope (Nikon, Tokyo, Japan) [24]. For immunohistochemistry, the sections were incubated overnight with rat anti-mouse CD68 (1:250; MCA1957GA; Bio-Rad, Contra Costa County, CA, USA) and rabbit anti-mouse IGF-1 (1:500; 40657; Abcam, Cambridge, UK) primary antibodies. Then, the samples were incubated for 30 min with Alexa Fluor 488-conjugated goat anti-rat immunoglobulin IgG (A11006; Invitrogen, Carlsbad, CA, USA) and Alexa Fluor 594-conjugated goat anti-rabbit IgG (A32740; Invitrogen, Carlsbad, CA, USA) secondary antibodies. Sections were mounted using the mounting medium containing 4′,6-diamidino-2-phenylindole (DAPI) (H-1200, Vector, Torrance, CA, USA). All sections were imaged using a confocal laser scanning microscope (FV10C-PSU; Olympus Corporation, Tokyo, Japan).

### 4.6. The Enzyme-Linked Immunosorbent Assay (ELISA)

TA muscle samples were harvested, lysed with PRO-PREP™ Protein Extraction Solution (17081; iNtRON Biotechnology, Jungwon, Korea) supplemented with a protease inhibitor cocktail, and then homogenized using a mechanical homogenizer (Precellys R 24; Bertin, Montigny-le-Bretonneux, France). ELISA kit for IGF-1 (DY791; R&D Systems, Minneapolis, MN, USA) was used according to the manufacturer’s instructions.

### 4.7. Quantitative PCR

Total RNA from TA muscles was harvested using easy-BLUE^TM^ Total RNA Extraction Kit (17061; iNtRON, Biotechnology, Jungwon, Korea). cDNA was generated using the Maxime RT-PCR PreMix Kit (25131; iNtRON Biotechnology, Jungwon, Korea). Gene expression levels were determined by real-time qPCR using the SensiFAST™ SYBR No-ROX Kit (BIO-98020; Medison bioline, Roma, Italia) and analyzed using the standard 2^−δδCt^ method and normalized to GAPDH (glyceraldehyde-3-phosphate dehydrogenase). *Nos2*(Forward: CAC CTT GGA GTT CAC CCA GT, Reverse: ACC ACT CGT ACT TGG GAT GC), *Gapdh*(Forward: ACC CAG AAG ACT GTG GAT GG, Reverse: CAC ATT GGG GGT AGG AAC AC), *Igf-1*(Forward: CTA CCA AAA TGA CCG CAC CT, Reverse: CAC GAA CTG AAG AGC ATC CA), *Tgfb1*(Forward: CAA GGA AGG TTG GCA TTT GT, Reverse: AGG TAA CGC CAG GAA TTG CA), *Tnfa*(Forward: GCT GAG CTC AAA CCC TGG TA, Reverse: CCG GAC TCC GCA AAGTCT AA), *Mrc1*(Forward: CAA GGA AGG TTG GCA TTT GT, Reverse: CCT TTC AGT CCT TTG CAA GC), *Arg-1*(Forward: GGC TGG TCT GCT TGA GAA AC, Reverse: CTT TTC CCA CAG ACC TTG GA), *Cd163*(Forward: TGG TGT GCA GGG AAT TAC AA, Reverse: ATC CCT GCT GTG GGT ACA AG).

### 4.8. Grip Test

All-limb or forelimb grip strength was measured using a digital force gauge (DS2-5N; IMADA Inc., Northbrook, IL, USA). The meter was placed horizontally for forelimb and all-limb tests. Mouse tails were pulled five times downwards to record the peak tension when the mice released their paws. All grip strength tests repeated thrice at 10 min intervals with an average of five values were used for calculations.

### 4.9. Statistical Analysis

Data are expressed as the mean ± standard error of the mean (SEM). The statistical analysis was performed using Prism 5.01 software (GraphPad Software Inc., San Diego, CA, USA). To compare each group of data from three independent experiments, a two-way ANOVA or a one-way ANOVA with a Bonferroni post hoc test were performed. Statistical significance was set at *p* < 0.05.

## 5. Conclusions

The M.C treatment alleviated cisplatin-induced sarcopenia by stimulating IGF-1 towards M2 macrophage. Our results suggest that treatment with M.C could significantly prevent cisplatin-induced sarcopenia in the mouse model.

## Data Availability

Data are contained within the article or Supplementary Materials.

## Supplementary Materials

The following are available online at https://www.mdpi.com/1422-0067/22/6/3188/s1.
