# Supplementary material for: Magnoliae Cortex Alleviates Muscle Wasting by Modulating M2 Macrophages in a Cisplatin-Induced Sarcopenia Mouse Model
Source: Int J Mol Sci. 2021 Mar 20;22(6):3188. doi: 10.3390/ijms22063188 (PMC8003985; doi:10.3390/ijms22063188)
Supplement: Supplementary file 1 [file ijms-22-03188-s001.pdf]

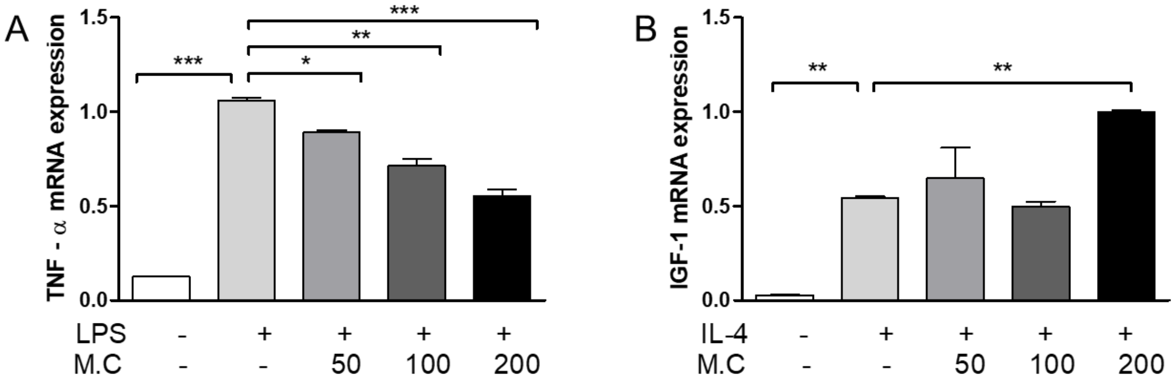

Supplementary Figure S1. Production of cytokines by RAW 264.7 macrophage cells. (**A**) RAW 264.7 cells were treated with LPS and M.C (0, 50, 100, 200 μg/ml) and incubated for 3 days; (**B**) RAW 264.7 cell were treated by IL-4 and M.C (0, 50, 100, 200 μg/ml) and incubated for 3 days. Cytokines mRNA were measured by using RT-qPCR. Data are presented as the mean ± SEM (n= 4/group). * $p < 0.05$, ** $p < 0.01$, *** $p < 0.001$, using Bonferroni post hoc test after one-way ANOVA.

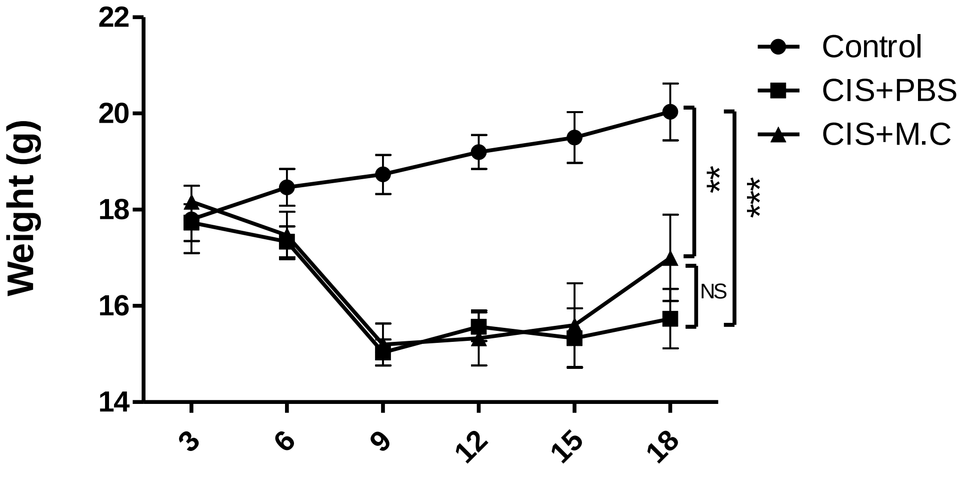

Supplementary Figure S2. Weight change after cisplatin and M.C treatment in colon cancer-bearing mice. CT26 tumor cells were injected subcutaneously into mice, and after 5 consecutive days, cisplatin (2.5 mg/kg, I.P) was injected, and they were treated with M.C (200 mg/kg, P.O) every 3 days, for a total of 6 times. Mice were sacrificed on day 18. Mice were measured every 3 days. Data are presented as the mean ± SEM (n = 5). * $p < 0.05$, ** $p < 0.01$, *** $p < 0.001$ vs. control, using a two-way ANOVA.
